# Supreme activity of gramicidin S against resistant, persistent and biofilm cells of staphylococci and enterococci

**DOI:** 10.1038/s41598-019-54212-z

**Published:** 2019-11-29

**Authors:** Marina Berditsch, Sergii Afonin, Jennifer Reuster, Hannah Lux, Kristina Schkolin, Oleg Babii, Dmytro S. Radchenko, Issah Abdullah, Nicola William, Volker Middel, Uwe Strähle, Andrew Nelson, Klara Valko, Anne S. Ulrich

**Affiliations:** 1 Karlsruhe Institute of Technology (KIT), Institute of Organic Chemistry (IOC), Karlsruhe, 76131 Germany; 2 KIT, Institute of Biological Interfaces (IBG-2), Karlsruhe, 76021 Germany; 3 Enamine Ltd., Kyiv, 02094 Ukraine; 4 Taras Shevchenko National University of Kyiv, Kyiv, 01601 Ukraine; 5 University College London (UCL), UCL School of Pharmacy, London, WC1N 1AX United Kingdom; 6 University of Leeds, School of Chemistry, Leeds, LS9 2JT United Kingdom; 7 KIT, Institute of Toxicology and Genetics (ITG), Eggenstein-Leopoldshafen, 76344 Germany

**Keywords:** Membrane biophysics, Antibiotics, Peptides

## Abstract

Three promising antibacterial peptides were studied with regard to their ability to inhibit the growth and kill the cells of clinical strains of *Staphylococcus aureus*, *Enterococcus faecalis* and *Enterococcus faecium*. The multifunctional gramicidin S (GS) was the most potent, compared to the membranotropic temporin L (TL), being more effective than the innate-defence regulator IDR-1018 (IDR). These activities, compared across 16 strains as minimal bactericidal and minimal inhibitory concentrations (MIC), are independent of bacterial resistance pattern, phenotype variations and/or biofilm-forming potency. For *S. aureus* strains, complete killing is accomplished by all peptides at 5 × MIC. For *E. faecalis* strains, only GS exhibits a rapid bactericidal effect at 5 × MIC, while TL and IDR require higher concentrations. The biofilm-preventing activities of all peptides against the six strains with the largest biofilm biomass were compared. GS demonstrates the lowest minimal biofilm inhibiting concentrations, whereas TL and IDR are consistently less effective. In mature biofilms, only GS completely kills the cells of all studied strains. We compare the physicochemical properties, membranolytic activities, model pharmacokinetics and eukaryotic toxicities of the peptides and explain the bactericidal, antipersister and antibiofilm activities of GS by its elevated stability, pronounced cell-penetration ability and effective utilization of multiple modes of antibacterial action.

## Introduction

Inappropriate use of antibiotics in medicine and agriculture has led to the emergence of resistant bacteria worldwide, endangering the clinical efficacy of conventional antibiotic regiments^1^. Among Gram-positive pathogens, a global pandemic of resistant *Staphylococcus aureus* and *Enterococcus* species currently poses the greatest threat^1^. Notably, both bacterial groups are among the major nosocomial opportunistic pathogens (ESKAPE group), for which the spread of multidrug-resistant (MDR) strains and high clinical relevance are well recognized^2^. In particular, staphylococci are a leading cause of bacteraemia and infective endocarditis as well as osteoarticular, skin/soft tissue, pleuropulmonary and device-related infections. Many staphylococci have become resistant to practically all of the commonly available agents. A notorious case is methicillin-resistant *S. aureus* (MRSA), perhaps the most studied example of the kind. Some strains of MRSA possess an additional resistance to aminoglycosides, macrolides, tetracyclines, amphenicols, lincosamides, rifampicin, daptomycin, mupirocin and fusidic acid^3–7^. Fortunately, due to the infrequency of horizontal gene transfer from vancomycin-resistant enterococci, only rare cases of completely vancomycin-resistant *S. aureus* (VRSA) isolates have been found^8^. In medical practice, enterococci are known to cause a wide range of clinical infections, from localized urinary tract infections and intra-abdominal infections to sepsis and endocarditis^9^. Enterococci are also a prominent cause of complex endodontic infections, including cases reported to be caused by tetracycline-resistant *E. faecalis* (TRE)^10^. *Enterococcus faecium* and *E. faecalis* are recognized causes of nosocomial infections and are ranked second (after *S. aureus* and *S. epidermidis*) as aetiological agents of hospital-associated infections^11^.

Besides genetic adaptation, bacterial resistance to antibiotics can also have a lifestyle-associated or phenotypic nature. This broad and intrinsic multidrug tolerance is often attributed either to the ability of bacterial cells to aggregate and adhere to surfaces, forming biofilms, or to the existence of small subpopulations of dormant persister cells within bacterial communities^12,13^. Biofilm-related or persisting infections are mostly chronic and require more intense care^14^. Biofilms are difficult to treat with antibiotics, because sessile cells are embedded in an extracellular, self-produced, complex matrix containing the polysaccharide intercellular adhesin (PIA), extracellular DNA, as well as various proteins, lipids and amyloid fibrils^15^. It has been suggested that the biofilm matrix can reduce or delay the infiltration of chemicals, including antibiotics, into the biomass^16^. In addition, biofilms represent a dangerous reservoir of persister cells, which can serve as a nidus of re-infection in the human body^17^. Remarkably, switching to the biofilm lifestyle may occur in planktonic bacteria as a response to an exposure to sub-inhibitory concentrations of antibiotics^18,19^. We have demonstrated that susceptible *S. aureus* and *E. faecalis* could sustain the exposure to some membrane-active peptides by switching into sessile growth mode^20^. Importantly, pretreatment with sub-lethal concentrations of antibiotics, irrespective of the growth mode, substantially increases the levels of persister cells – phenotypic “surviving” cells, which do not experience any genetic alterations^21,22^. Among other proposed mechanisms, transcriptome analysis of some isolated persisters suggested toxin-antitoxin modules as important controllers of persister formation^17,23^. The function of several such modules was shown to be regulated by the bacterial stress alarmon (p)ppGpp^24^. It is believed that transitioning into metabolically dormant persisters allows bacteria to tolerate antibiotics simply due to the multiplicity of inactive targets^25^.

Frequent isolation of slow-growing clinical pathogens from biofilm-associated infections, can be regarded as a medical manifestation of bacterial phenotype switching. These isolates are subpopulations described as small-colony variants (SCVs), i.e. forming colonies only one-tenth the size of the common phenotype on agar plates^26^. Although SCVs have been described for many genera of bacteria, they have been studied most extensively in staphylococci^25,26^. The often transient nature of SCVs suggests that they represent a part of the normal life cycle^27^. Interestingly, an increased biofilm-forming capacity has been reported for many SCVs^13^.

To combat pathogens with acquired resistance, elevated persistence and/or high biofilm-forming capacity, new treatment approaches using a broad spectrum of alternatives have been suggested^28^. Among them are antibodies, probiotics, bacteriophages, immunostimulants, vaccines, and antimicrobial peptides (AMPs). The advantages of antimicrobial peptides are their rapid bactericidal action, low target-based resistance, and low immunogenicity^28^. However, due to poor cell selectivity, resulting in highly unwanted host toxicity, and an intrinsic lability to proteases, they are generally not used systemically, although in some instances a topical application may effectively supplement systemic therapy^28,29^. The vast majority of antimicrobial peptides are large molecules, resulting in significant production costs and poor pharmacokinetic properties^30^. Therefore, our particular interest was in short sequences that can be efficiently made by chemical synthesis or produced by bacterial fermentation at competitive costs^31^. In this study, we aimed to systematically compare the well-known cyclic decapeptide gramicidin S (GS: cyclo[fPVOL]_2_, f = *D*-phenylalanine, O = Ornithine) of bacterial origin with two other promising peptides, temporin L (TL: FVQWFSKFLGRIL-amide) and IDR-1018 (IDR: VRLIVAVRIWRR-amide)^29,32–38^. The naturally occurring linear tridecapeptide TL was originally found in the skin of *Rana temporaria*^35^. Its antimicrobial activity is more highly expressed towards Gram-positive bacteria than Gram-negative bacteria^36^. The antibiofilm properties of TL have not been studied. The synthetic linear dodecapeptide “innate defense regulator” IDR, was derived by substantial modification of the host-defence peptide bactenecin from bovine neutrophils. Its specific antibiofilm activity has been intensively studied in the last decade^37,38^. Herein, we have examined the antibacterial activities of the three peptides towards various relevant clinical strains, including MDR *S. aureus*, *E. faecalis* and *E. faecium*, stable and transient SCVs of *S. aureus*, and strains with elevated biofilm-forming capacity.

## Results

### Characterization of peptides

The peptides were synthesized by standard solid-phase peptide synthesis protocols. Chemical synthesis of GS requires an additional step of cyclization in dilute solution, following the cleavage of the linear construct from the resin. Hence, despite having the smallest number of amino acids, GS synthesis is more demanding than the production of linear TL and IDR. Luckily, GS is readily available by bacterial fermentation. As it undergoes the same purification steps (high-performance liquid chromatography, HPLC) as the other two peptides, irrespective of the production route, its biosynthetic production should be preferred. Indeed, when we compared the activities of synthetic and biosynthetically produced GS, we observed no difference between the two. In the following, only biosynthetic GS was used to avoid batch-to-batch uncertainties.

**Figure 1:**
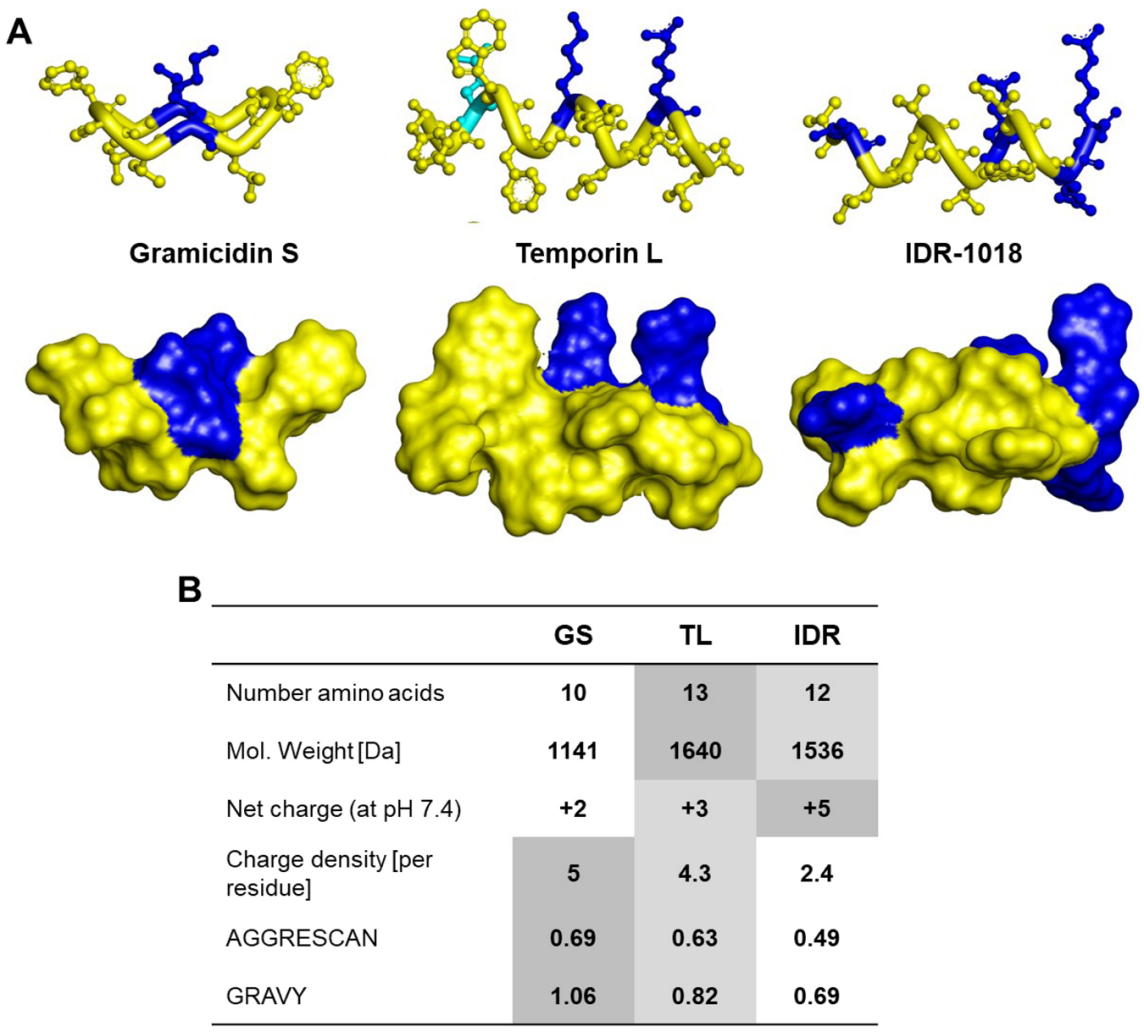
Structural and compositional properties of the studied peptides. (**A**) Molecular models of the active conformations highlighting the distribution of hydrophobic (yellow), polar (cyan) and cationic (blue) residues. (**B**) Theoretical properties of the peptides. AGGRESCAN values were calculated according to reference^78^ and represent an average of the amino acid aggregation propensities over the sequence; GRAVY values were calculated as a sequence average of hydropathy values^79^. For both calculations, the lysine values were used to characterize non-canonical ornithine. The highest absolute values of all parameters are highlighted with a dark gray and the lowest with a white background color.

All three peptides are short (less than 13 residues), amphipathic, and hence are active against lipid bilayers. They carry a net cationic charge, thus possessing an electrostatically mediated selectivity towards anionic membranes. They are composed of different types of positively charged amino acids, however, (2 non-canonical ornithines in GS, 1 Agr & 1 Lys in TL, and 4 Arg in IDR) and vary in charge density, besides the obvious differences in secondary structures. Figure 1 shows molecular models of their functionally relevant conformations and summarizes the predicted physicochemical properties.

The decameric GS has a symmetric cyclic structure that is rather compact. The two linear peptides are larger in size, and IDR is the most highly charged, which results in its lowest absolute hydrophobicity and lowest ability to aggregate. A principal difference between the three peptides obviously lies in their conformational propensities. In contrast to GS, which maintains a largely constant structure independent of the environment, TL and IDR are linear peptidylamides and hence possess much higher conformational plasticity^39–41^. Indeed, TL and IDR are unfolded in aqueous solvents, and TL folds into an α-helix only in membrane-mimicking environments, whereas IDR in the presence of membrane models can fold either as an α-helix or as a β-turn.

**Figure 2:**
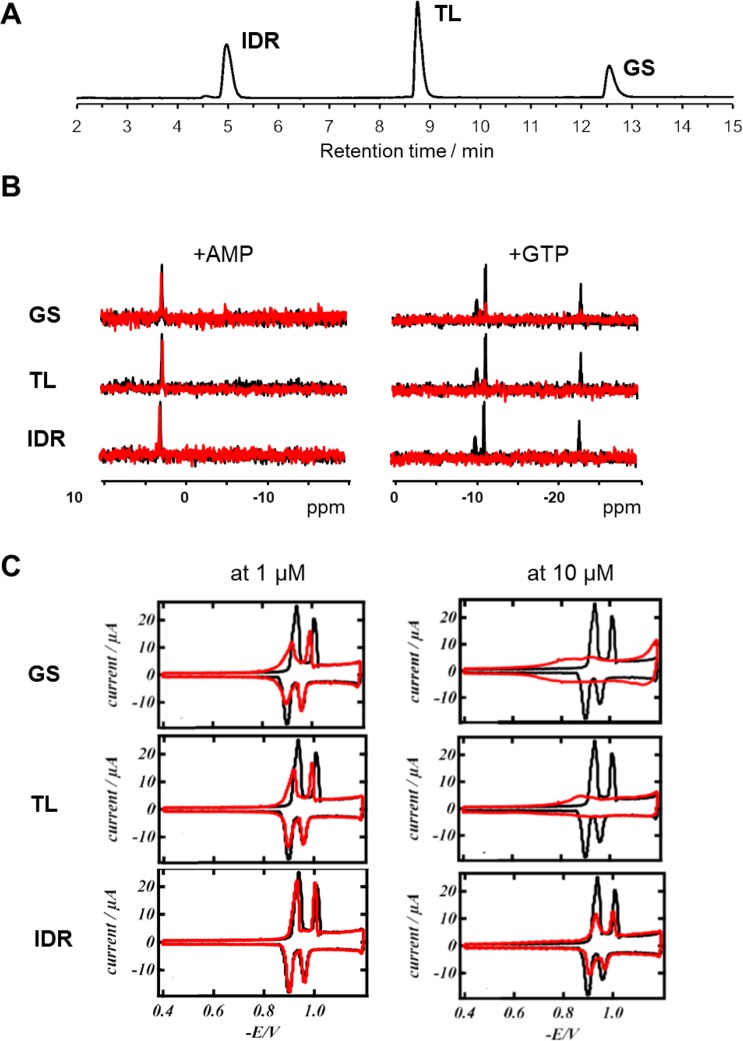
Representative readouts illustrating the experimentally determined differences between the peptides in the following. (**A**) chromatographic behaviour of the peptide mixture in analytical HPLC using a water/acetonitrile gradient and C_18_ stationary phase; (**B**) interactions with soluble phosphate-containing anions observed by ^31^P-NMR spectroscopy using equimolar mixtures of the peptides with P-metabolites, where no interaction (e.g., AMP, left) and strong interaction (e.g., GTP, right) could be demonstrated (black spectra show controls without peptide; red spectra show the results upon addition of the peptides); (**C**) ability to disturb zwitterionic lipid monolayers by rapid cyclic voltametry (black traces – RCV plots of pure DOPC on the Hg/Pt electrodes; red traces – RCV plots in the presence of different peptide concentrations).

Compositional differences translate into functional properties, as observed in the physicochemical experiments addressing the peptides´ mechanisms of action (Fig. 2). Determined under reversed-phase chromatography on a standard C_18_ column, the apparent hydrophobicity in the partially folded state is in the order IDR < TL < GS, corroborating the AGGRESCAN values. The ability to perturb supported zwitterionic lipid monolayers as established models^42^ for bilayers was measured by rapid cyclic voltammetry (RCV) and correlates best with the charge densities, with IDR being the weakest monolayer perturbant (IDR«TL < GS). The same outstanding electrostatic-driven binding by IDR was observed in anion-binding studies (Fig. 2B). Here, we compared the interactions of the 3 AMPs with various phosphate-containing cellular metabolites (P-metabolites), by measuring ^31^P-NMR spectra in equimolar P-metabolite/AMP aqueous solution. Judging by the disappearance of the signals (often with visible precipitate formation) and/or modulation of the chemical shifts, we could distinguish two types of interaction in the binary mixtures – (i) no binding, and (ii) ^31^P-NMR-detected interactions. Most P-metabolites - AMP, ADP, GDP, phosphoenolpyruvate, a short-chain phosphatidylcholine (1,2-dihexanoyl-*sn*-glycero-3 phosphocholine) and monophosphate - were not influenced by the presence of the peptides, whereas disctinct interactions with ATP, GTP, pyrophosphate, butyryl phosphate and ppGpp suggested a molecular basis for an interference of the peptides with the energy metabolism in bacterial cells. In all cases, IDR was found to be the strongest in binding, followed by TL and GS, which resemble the order in which the charge density decays.

**Figure 3:**
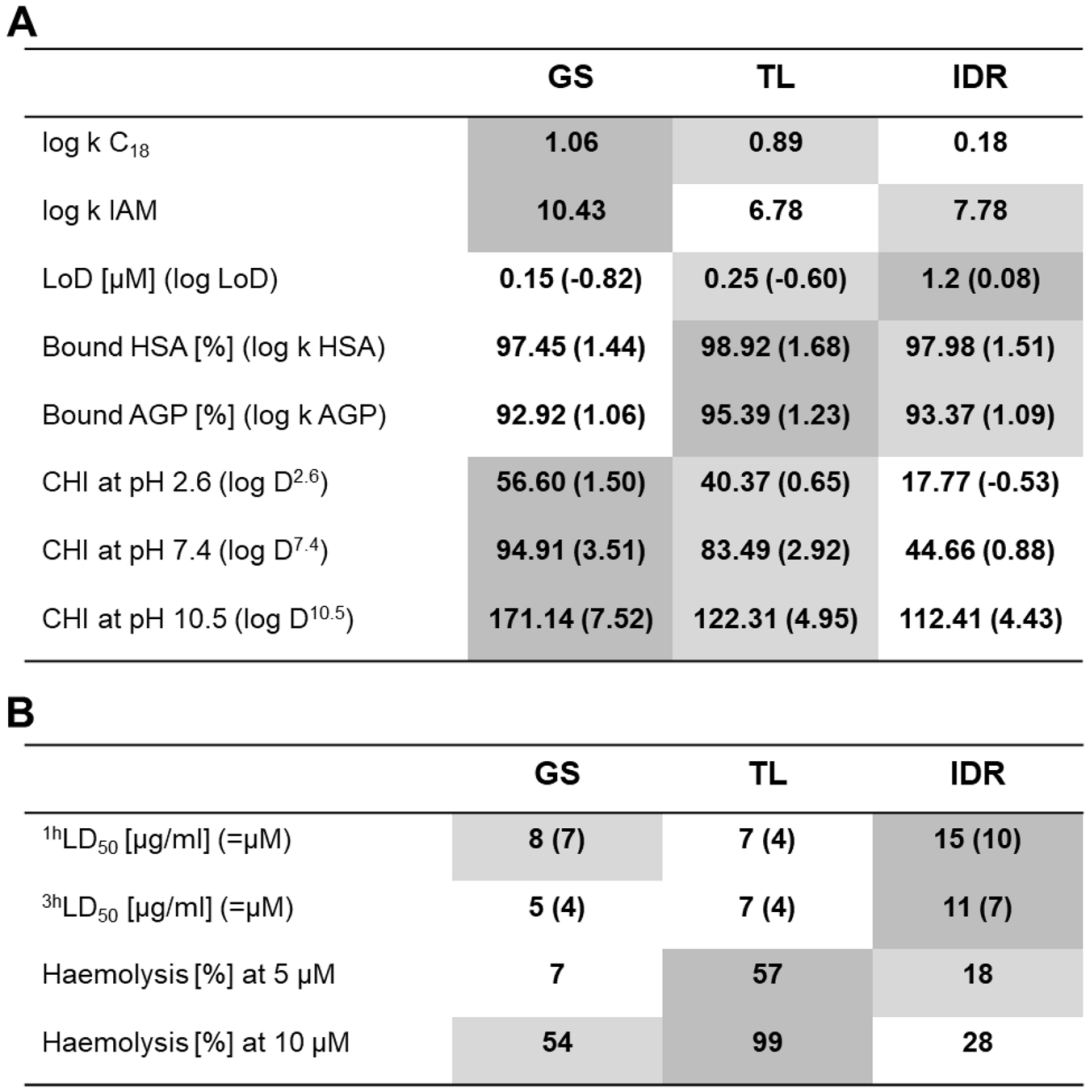
Quantitative comparison of the experimental physicochemical and toxicity properties of the peptides. (**A**) General hydrophobicity (log k C_18_); binding to immobilized 1,2-dimyristoyl-sn-glycero-3-phosphocholine (log k IAM); ability to perturb DOPC bilayer (LoD, log LoD); binding to HSA (binding, log k HSA); binding to AGP (binding, log k AGP); and hydrophobicity under acidic, basic and neutral conditions (CHI pH, log D^pH^). (**B**) *In vivo* toxicity (LD_50_) against zebrafish larvae after 1 hour and 3 hours of exposure and human erythrocyte haemolysis at different concentrations. The highest absolute values of all parameters are highlighted with dark gray and lowest with a white background color.

We further compared the model pharmacokinetic parameters of the peptides by measuring their response to biomimetic chromatography conditions. As summarized in Fig. 3, the chromatographic hydrophobicity indices (CHIs) of the peptides, irrespective of the ionizing conditions, always revealed the same order IDR < TL < GS and showed corresponding binding to the C_18_ stationary phase in reversed-phase HPLC (Fig. 2A). However, the CHI values failed to explain the differential binding to immobilized artificial membranes (IAM). In IAM chromatography, where the peptides are partitioning into phosphatidylcholine monolayers (the immobilized lipid being 1,2-dimyristoyl-*sn*-glycero-3 phosphocholine), GS showed again the strongest hydrophobic interaction, but TL-binding was significantly weaker than IDR. Interestingly, when the peptides were exposed to other species of phosphatidylcholines, the rankings of the ability to bind lipids were different. We thus note the obviously strong membrane-perturbing abilities of TL shown in the experiments exploiting DOPC (1,2-dioleoyl-*sn*-glycero 3-phosphocholine) as a membrane model (see LoD (=limit of detection) values and RCV plots (Figs. 2C, 3A), and the apparent inability of all AMPs to bind 1,2-dihexanoyl-*sn*-glycero-3-phosphocholine in solution (see ^31^P-NMR results). This discrepancy can be resolved if we consider the lasting prevalence of the hydrophobic binding forces over the initial electrostatics-mediated attraction to be the major determinants of the peptide-membrane interactions. Accordingly, TL and GS both could be suggested as immersing deeper into the apolar core of the outer bilayer leaflet to reveal their membranolytic action. Interestingly, the ability to bind to acidic proteins - water-soluble human serum albumin (HSA, isoelectric point ~4.7) and to α1-acid glycoprotein (AGP, isoelectric point ~3.3) must include hydrophobic contributions from the net cationic peptides, as for both parameters the most highly charged IDR revealed only intermediate binding values. Nontheless, all three peptides were found to be >90% bound when exposed to HSA- or AGP-immobilized chromatography columns. This high nonspecific binding should additionally influence the *in vivo* pharmacodynamics of all three AMPs, making it tremendously difficult to maintain high bolus blood concentrations. On the other hand, systemic toxicity will also decay correspondingly.

Additional pharmacologically relevant differences in the action of AMPs were evident in the evaluation of eukaryotic toxicity using various assays (Fig. 3B). *In vivo*, all three peptides were shown to be acutely toxic. Upon extracorporeal application in a zebrafish embryotoxicity model, LD_50_ values (50% lethality dose) in the 4–10 µM range were consistently found. Interestingly, neither the order of toxicity (e.g. IDR < TL~GS for 3 h exposure LD_50_, vs. GS < IDR < TL at 5 µM in the haemolysis assay), nor the absolute toxicity levels correlated between the two toxicity assays (IDR and GS taken at LD_50_ concentrations did not show significant haemolysis). This finding suggests that in our *in vivo* experiments, haemolysis was not the major lethality factor, but other toxicity mechanisms could and should have contributed. We also noted that the kinetics of *in vivo* toxicity varied between the peptides. TL was the only peptide where LD_50_ did not change between 1 hour and 3 hours of exposure, whereas IDR and GS killed more embryos at longer incubation times. This result corroborates the anticipated proteolytic stability of the three AMPs. TL should be the most labile - it contains a canonical trypsin cleavage site (Lys-Phe); IDR should be intermediate as a folded bactenecin analogue^43^; and GS, due to its cyclic nature and the presence of non-canonical amino acids, is essentially not susceptible to proteolysis.

### Characterization of bacterial strains

Prior to systematically addressing the antibacterial properties of the peptides, we characterized the resistance patterns of the available strains. The resistance of clinical isolates to conventional antibiotics was determined in the original laboratories, using the VITEK-2 system. Additionally, we tested all staphylococci against mupirocin^6^, and the control strain *S. aureus* DSM 1104 and its SCV towards methicillin, oxacillin (OXA), gentamicin, tetracycline, and streptomycin, using the standard broth microdilution procedure^44^. We also verified the MICs of demeclocycline (DMC), vancomycin and gentamicin against the control strain *E. faecalis* DSM 2570, and *E. faecalis* isolates WW4 and WW6. The results are summarized in Supplementary Information (SI), Table S1. The MDR strains, classified as possessing resistance to at least one agent in three or more antimicrobial categories^45^, were *S. aureus* MRSA9, MRSA9 SCV, MRSA538 SCV, all *E. faecalis* TRE, and *E. faecium* VRE strains.

**Figure 4:**
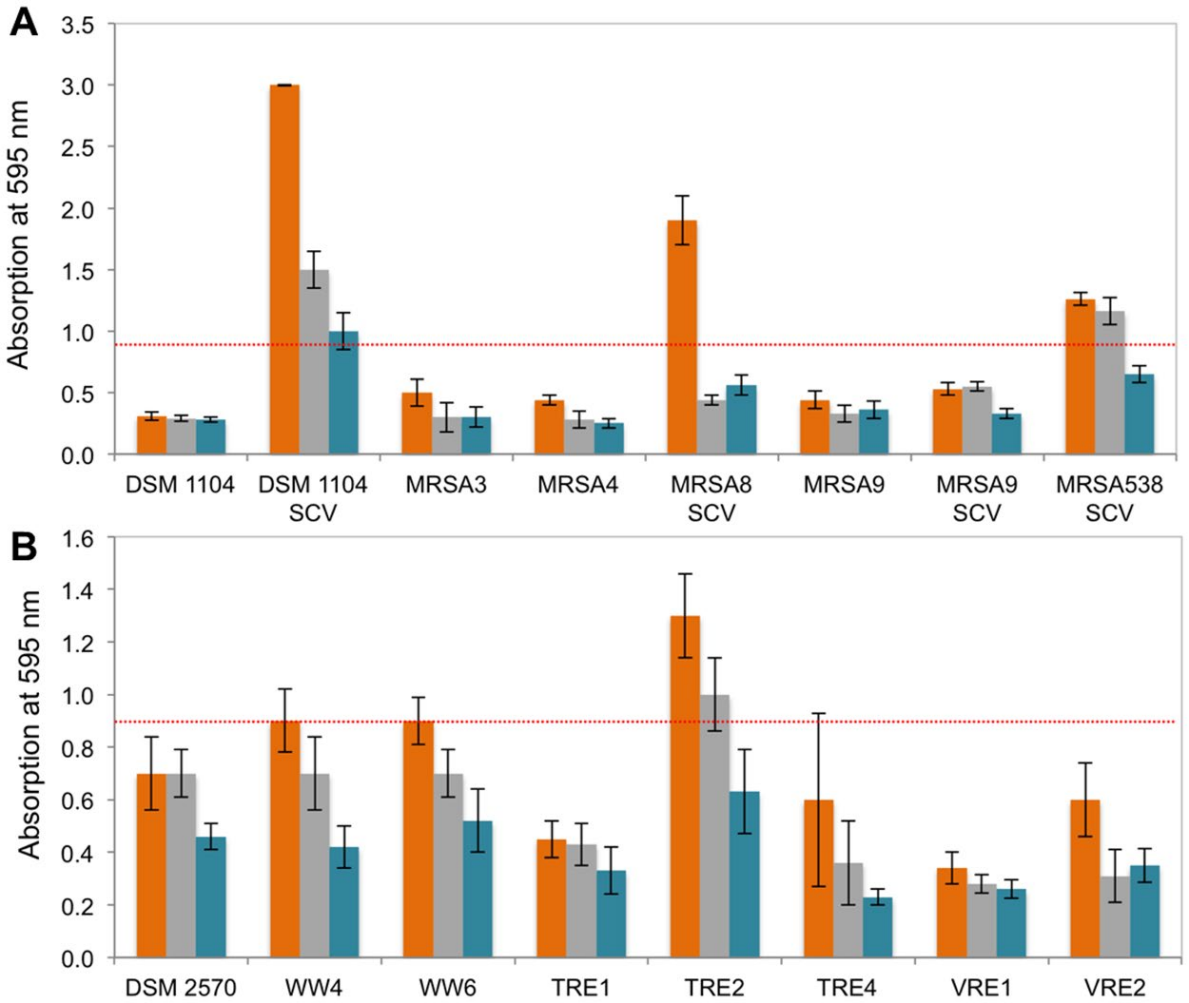
Biofilm-forming capacity in three different growth media. (**A**) *S. aureus* strains, (**B**) *E. faecalis* and *E. faecium* strains. Orange – when being cultured in TH broth, gray – in MH broth, teal – in MM. Dotted red lines represent the level of Crystal violet absorption = 0.9.

Next, we analysed all 16 strains for their biofilm-forming abilities in different nutrient media: a Todd-Hewitt (TH) broth, a Mueller-Hinton (MH) broth, and a minimal medium (MM); the latter had been applied previously for the study of biofilm eradication^38^. The purpose of this examination was to determine the conditions that promoted growth with the largest biofilm biomass, and to select the most potent biofilm-forming strains for analyzing the antibiofilm activities of the peptides. Our results (Fig. 4) indicate that cultivation in TH broth is uniformly the most appropariate condition that consistently enables the growth of robust biofilms for all bacteria. In the first 24 hours, vigorous biofilm development was observed among *S. aureus* for DSM 1104 SCV, MRSA538 SCV, and MRSA8 SCV, but not for MRSA9 SCV. The latter isolate grew slowly and, in the conditions of a 96-well microtiter plate, required at least 48 hours to complete biofilm development (data not shown). The strongest biofilm-forming *E. faecalis* strains were TRE2, WW4, and WW6, among which only TRE2 is an MDR strain (SI Table S1).

We further analysed the stability of the available *S. aureus* SCVs. Their reversion into the classical large colony variants (LCVs) was defined by streaking of the overnight cultures onto TH agar. As shown in SI Fig. S1, the appearance of LCVs in the cultures of DSM 1104 SCV and MRSA9 SCV clearly suggests the transient nature of both variants. Additionally, DSM 1104 SCV develops distinct agglomerates in the first hours of growth in the liquid culture, followed by passage into fully planktonic growth only after 22 hours of cultivation (SI Fig. S2). This observation clearly highlights a unique biofilm-forming ability of DSM 1104 SCV even in liquid medium. The remaining *S. aureus* SCVs grew planktonic when in liquid cultures. MRSA8 SCV and MRSA538 SCV both appeared to be stable SCV forms, as they did not revert into LCV (data not shown).

We analysed the peculiarities of the biofilm matrix composition in the transient *S. aureus* SCVs (DSM 1104 SCV and MRSA9 SCV), their parental variants, and one stable SCV (MRSA8 SCV) by Congo red staining. The interaction with Congo red was studied in two different assays: bacteria were grown as biofilms on hydroxyapatite discs (HAD), and as colonies on brain heart infusion agar plates (SI Fig. S3). A distinct black colour suggested the elevated production of PIA by both phenotypic variants of DSM 1104, but not by any of the tested MRSA strains. These data corroborate previously described biofilm phenotypes of the MSSA strains, which are highly enriched in PIA^46^. Interestingly, the ability to synthesize PIA does not provide DSM 1104 variants with adaptive tolerance advantages, as planktonic cells remain sensitive to the action of antibiotics (SI Table S1).

### Minimum inhibitory and bactericidal concentrations

Next, we analysed the influence of the peptides on all bacterial strains by determining the MIC and minimum bactericidal concentration (MBC) values.

Comparison of the three peptides consistently reveals the best overall antimicrobial activity for GS and the worst for IDR (Table 1). The MIC values of GS against *S. aureus* were mostly 4 µg/ml, only for enterococci they were seemingly one dilution higher, approximately 8 µg/ml. The same selectivity trend could be suggested for TL. Interestingly, the opposite tendency was apparent for IDR, which was overall more active against enterococci. Nevertheless, except for the activity against VRE2, IDR showed the highest MIC values, indicating its globally lower antimicrobial activity. Although the MIC values of all three peptides were similar for two clinical *E. faecium* strains, GS clearly had the lowest MBC values.

**Table 1:** Minimum Inhibitory and Bactericidal Concentrations of GS, TL and IDR.

Strain	MIC [µg/ml] ( = µM)			MBC [µg/ml] ( = µM)		
	GS	TL	IDR	GS	TL	IDR
*S. aureus*						
DSM 1104	4 (4)	8 (5)	32 (21)	8 (7)	16 (10)	128 (83)
DSM 1104 SCV	4 (4)	8 (5)	64 (42)	8 (7)	16 (10)	128 (83)
MRSA3	4 (4)	8 (5)	64 (42)	16 (14)	32 (20)	128 (83)
MRSA4	4 (4)	8 (5)	32 (21)	16 (14)	32 (20)	> 256
MRSA8 SCV	4 (4)	8 (5)	64 (42)	16 (14)	16 (10)	128 (83)
MRSA9	4 (4)	8 (5)	32 (21)	16 (14)	32 (20)	128 (83)
MRSA9 SCV	4 (4)	8 (5)	64 (42)	16 (14)	32 (20)	128 (83)
MRSA538 SCV	8 (7)	16 (10)	64 (42)	16 (14)	32 (20)	128 (83)
*E. faecalis*						
DSM 2570	8 (7)	16 (10)	32 (21)	8 (7)	16 (10)	64 (42)
WW4	8 (7)	16 (10)	32 (21)	16 (14)	32 (20)	64 (42)
WW6	8 (7)	16 (10)	32 (21)	16 (14)	32 (20)	64 (42)
TRE1	8 (7)	16 (10)	32 (21)	16 (14)	32 (20)	64 (42)
TRE2	16 (16)	16 (10)	32 (21)	16 (14)	32 (20)	128 (83)
TRE4	8 (7)	16 (10)	16 (10)	8 (7)	32 (20)	16 (10)
*E. faecium*						
VRE1	8 (7)	8 (5)	16 (10)	8 (7)	16 (10)	32 (21)
VRE2	8 (7)	8 (5)	8 (5)	8 (7)	16 (10)	32 (21)

### Time- and concentration-dependent killing effect of peptides

**Figure 5:**
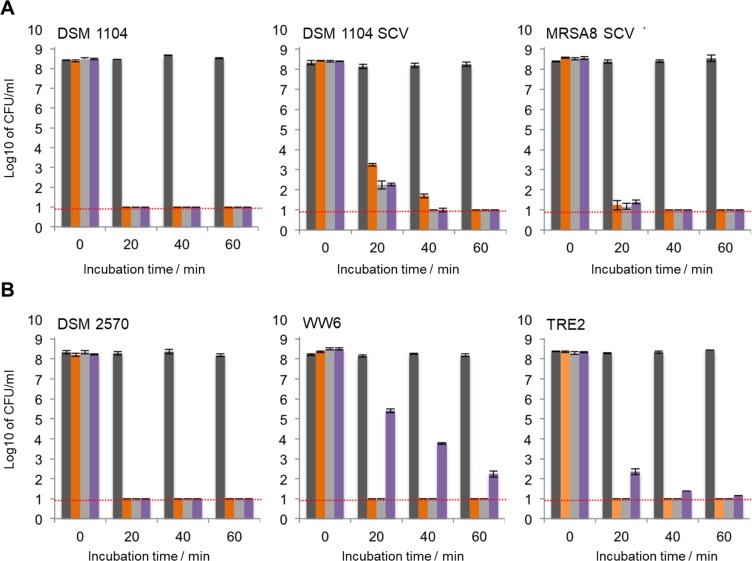
Reduction of the bacterial cell number during peptide treatment. (**A**) *S. aureus* strains treated at 5 × MIC. (**B**) *E. faecalis* strains treated at 10 × MIC. Dark gray bars are from the control (no peptide), and cell counts are shown for incubation with GS (orange), TL (light gray) and IDR (purple). The LoD was 10^1^ CFU/ml (red lines).

To characterize the killing kinetics, we analysed the exposure of 10^8^ CFU/ml (CFU = colony-forming units) planktonic bacteria to supra-MIC concentrations and monitored the cell number as a function of time. With all three peptides, when exposed to 5 × MIC, *S. aureus* counts dropped to 10^1^ CFU/ml (LoD) in less than one hour (Fig. 5A). However, the same conditions were less effective against the cells of *E. faecalis* (SI Fig. S5). Whereas GS at 5 × MIC accomplished killing within 60 min in all cases, TL showed comparable effectiveness only against DSM 2570 and TRE2 but was not able to eliminate WW6. IDR at 5 × MIC (160 µg/ml) was the worst in performance: it killed only *E. faecalis* TRE2 cells and required the full 60 min. The cells of the other two strains, though reduced in numbers, remained viable (SI Fig. S5). At 10 × MIC (Fig. 5B), GS (80 µg/ml) and TL (160 µg/ml) were again able to kill all *E. faecalis* cells within 20 min. IDR (320 µg/ml) was similarly effective against DSM 2570, exhibited slower killing of TRE2, and could not complete its action against WW6 planktonic cells within 60 min of incubation.

In all cases, we observed a monophasic killing process. At sufficiently high concentration, rapid and complete killing by all antimicrobial peptides suggests that they are effective against persister cells. The results of the experiments are also consistent with the MIC/MBC evaluations, collectively allowing the peptides to be ranked as GS > TL»IDR in terms of effective bactericidal action against planktonic staphylococci and enterococci.

### Minimum biofilm inhibitory concentrations

**Figure 6:**
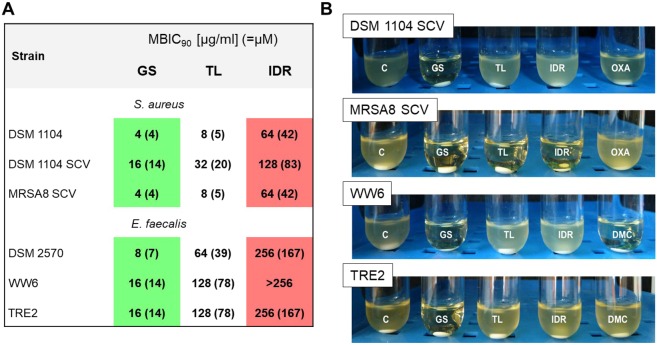
Antibiofilm activities of the peptides. (**A**) Minimum biofilm inhibitory concentrations towards the strongest biofilm-building strains of staphylococci and enterococci. The highest absolute values of all parameters are highlighted with red and lowest with a green background color. (**B**) Biofilm regrowth after treatment with 400 µg/ml peptides or conventional antibiotics. Control (no peptide) is designated “C”.

The MBIC_90_ (MBIC_90_ = minimal peptide concentration at which a bacterial strain develops <10% of the biofilm biomass of the untreated control) values for GS, TL and IDR were determined using a two-fold microdilution procedure, exploiting the best biofilm-forming strains identified above (Fig. 4). This parameter quantifies the ability of the peptides to prevent biofilm outgrowth. As summarized in Fig. 6A, GS was again the most effective.

### Biofilms on hydroxyapatite discs: regrowth and scanning electron microscopy

To characterize the viability of the biofilm cells, HAD with pregrown biofilms were exposed for 18 hours to supra-MBIC_90_ concentrations of the AMP, and - after washing - were placed into fresh TH broth. This procedure serves to determine possible planktonic regrowth from the cells that survived the treatment. The mature extended PIA-enriched biofilms of DSM 1104 SCV were tolerant not only to TL and IDR, but also to OXA (Fig. 6B), which was effective against the planktonic cells at 0.25 µg/ml (SI Table S1). At the same time, the complete lack of regrowth of these biofilms after exposure to GS reflects the excellent effectiveness for this particular antibiotic (Fig. 6B). The presence of GS abolishes regrowth for both antibiotic-susceptible *E. faecalis* WW6 and MDR *E. faecalis* TRE2 biofilms.

**Figure 7:**
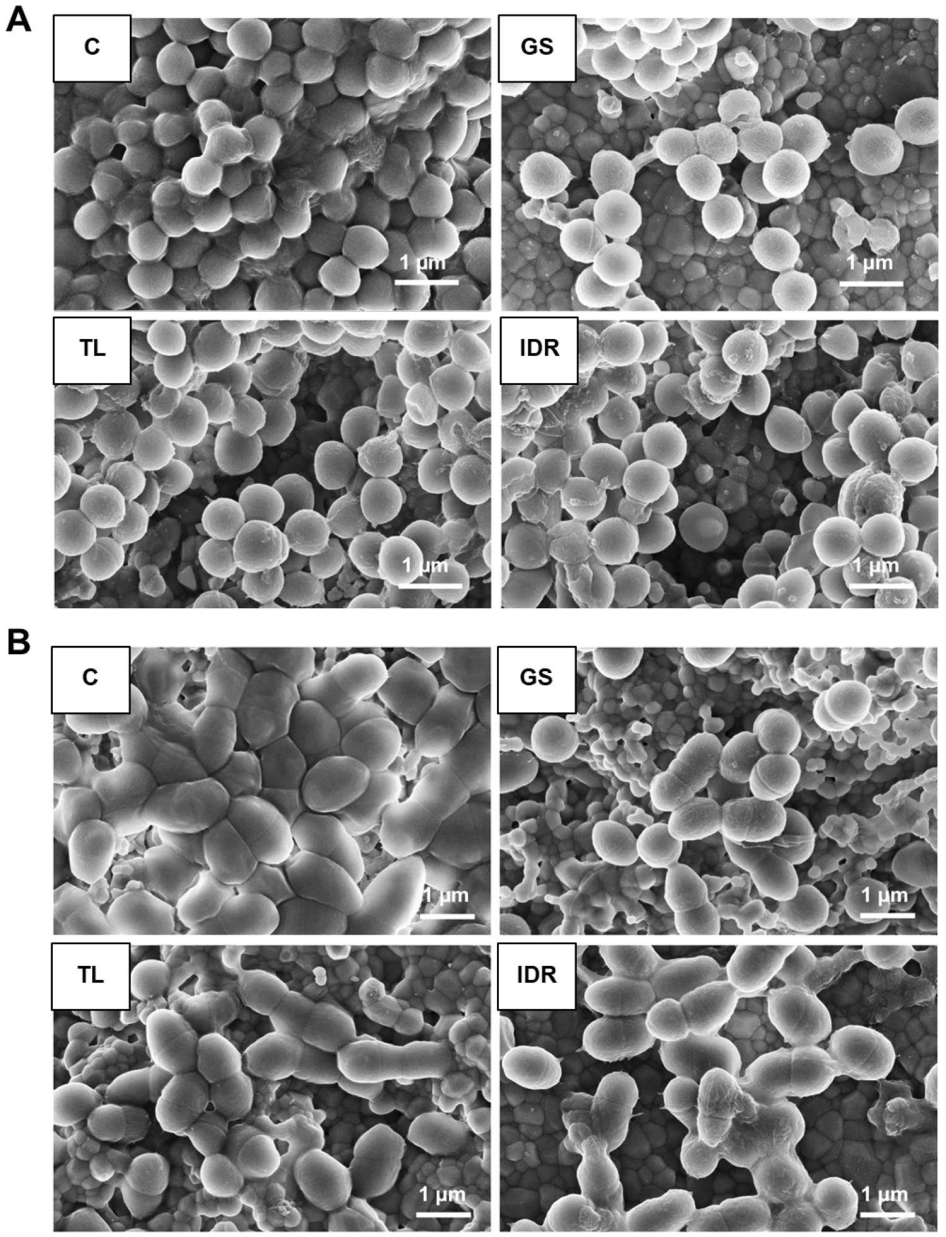
SEM images of biofilms grown on hydroxyapatite surfaces. (**A**) Cells of DSM 1104 SCV and (**B**) TRE2 biofilms before (C, control) and after treatment with GS, TL and IDR. The magnification of all images was 15.000 × .

The other two peptides were effective only against the weakest biofilm-former MRSA8 SCV. The surface-attached biofilms were also examined by scanning electron microscopy (SEM) to identify potential morphologic changes after peptide treatment. Without any peptide the SEM of the PIA-producing DSM 1104 SCV biofilms (Fig. 7A) revealed an external layer, which covered the cells and kept them together. This feature did not look like a soft gel, but rather resembled a glazed layer, as is often observed for polysaccharides. The PIA in the biofilm of DSM 1104 SCV obviously protected the cells from OXA, TL and IDR, but failed against GS action. This finding correlates with the reduced binding affinities of GS to proteins and glycoproteins described above (Fig. 3A). In contrast, the adhesion in the PIA-poor biofilms of MRSA8 SCV was observed to be mediated by surface proteins and unidentified fibrils (SI Fig. S6). The absence of PIA could be a prime reason for the high susceptibility of surface-grown MRSA8 SCV biofilms to the action of all three peptides.

In *S. aureus*, after exposure to AMPs, the extracellular matrix appeared significantly reduced, suggesting a mechanism for biofilm dispersion (Fig. 7). Notably, GS treatment not only caused the highest degree of matrix reduction and the lowest number of remaining cells, but also resulted in profound alterations in the morphology of the remaining cells. Their surfaces appeared wrinkled, many cells were swollen, and some were clearly disrupted. Such alterations were less pronounced upon exposure to TL and IDR.

Intact *E. faecalis* TRE2 biofilms were observed as layers of cells with a diameter of about 1 µm, seemingly covered by an unknown cell-associated material (Fig. 7B). After GS treatment, this material also disappeared, as could be judged from the perceived cell size reduction to about 0.8–0.9 µm. Single and dividing cells no longer appeared to be attached together, indicating a dispersion of the biofilm, as in the case of *S. aureus*. Here, again, TL and IDR exhibited poorer dispersion levels and influenced the remaining matrix to a lesser extent. More matrix continued to cover and adjoin the remaining cells, which stayed within distinct slime-enclosed aggregates. Many cells in both *S. aureus* DSM 1104 SCV and *E. faecalis* TRE2 biofilms, after co-incubation with TL and IDR, appeared healthy, which correlates with the higher MBIC_90_ values and positive regrowth results of these peptides. Notably, none of the peptides was able to eradicate the pregrown biofilms completely from the HAD.

## Discussion

Among the three peptides compared here, TL and GS are traditionally regarded as amphipathic membranolytic AMPs, whereas IDR was originally highlighted as a specific biofilm-targeting peptide. IDR was credited with the unique feature of binding to the intracellular alarmon (p)ppGpp, hence offering a new tool to combat the general drug tolerance of biofilms. IDR/alarmon interactions were proposed as a universal biofilm-preventing mechanism in multiple Gram-negative and Gram-positive pathogens^38,47^. *In vivo*, however, IDR has also been reported to differentially accelerate wound healing in several *S. aureus*-infected porcine and murine models. Notably, these effects were not related to the impact of IDR on bacterial colonization levels and could be explained by a modulation of the host response to bacterial challenge, rather than by any specific antibiofilm activity^48^. Unfortunately, another study failed to confirm the preferential activity of IDR against biofilms of *Pseudomnas aeruginosa*^49^. Our results further corroborate these later findings, revealing the low potency of IDR in preventing the biofilm growth of *S. aureus* and *E. faecalis* as well as its inability to eliminate pregrown biofilms of *S. aureus* DSM 1104 SCV, *E. faecalis* TRE2 and WW6. Moreover, it appears that in a broader context of molecular interactions with anionic phosphate-containing metabolites, all three peptides are equally selective in binding. In equimolar mixtures, quantitative differences in electrostatic binding may be observed (Fig. 2B), but they can be readily explained by the higher net positive charge of IDR (Fig. 1).

Here, in the *in vitro* settings, we demonstrate GS and TL to be *de facto* more effective than IDR in antibacterial actions against planktonic staphylococci and enterococci (Table 1), as well as in genuine biofilm-preventing (Fig. 6A) and biofilm-eliminating activities (Figs. 6B, 7) against numerous representative strains. Our comparative studies showed that GS, TL and IDR are equally effective against regular and persister cells, but the former two peptides are superior in the operative killing of planktonic cells at supra-MIC (Fig. 5). The physico-chemical ability of GS and TL to insert more deeply into the hydrophobic lipid bilayer core and cause stronger bilayer perturbations (Fig. 2C) readily explains their superiority and low selectivity, similar to that of other known membranotropic molecules^39,50–55^. Taken together with the surprisingly high eukaryotic toxicity of IDR (Fig. 3B), in medical practice the “canonical” antimicrobial membrane-active peptides, such as GS and TL, should be more applicable. Herein, despite the easier handling of TL due to its lower hydrophobicity, GS is definitely the more preferable choice.

Remarkably, only GS but not TL was effective towards all four studied pregrown biofilms, including the well-developed biofilm of *S. aureus* DSM 1104 SCV, the single-cell layer biofilm of MRSA8 SCV, and the biofilms of both selected *E. faecalis* strains (Fig. 6). According to our results, only GS is able to eradicate biofilms with an enhanced PIA-matrix and prevent subsequent regrowth. For such dense three-dimensional biofilms, TL and IDR, presumably, will require even higher concentrations or much longer times to be equally successful. Overall, the activity towards resistant, persistent and biofilm cells of clinical staphylococci and enterococci were found to correlate with the hydrophobicity of the peptides: the most hydrophobic peptide, GS, which possesses only two positive charges, showed the best activity in all studies.

Historically, GS was isolated from soil bacilli in the early 1940s and was one of the first peptides applied for the treatment of infected burns and wounds^32,56^. Eventhough the successful use of penicillin at that time led to a neglect of GS as an antiinfective topical drug, it is currently still being applied. Today, GS is available in Russia as a bactericidal agent against sore throats and mouth ulcers in the form of lozenges and the spray Grammidin^TM^.

**Figure 8:**
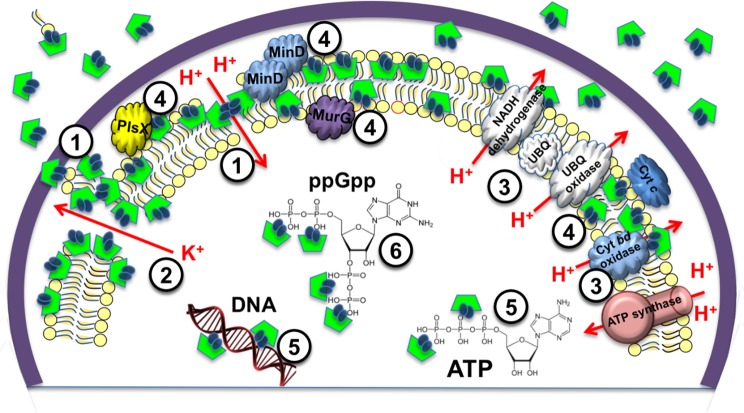
Multiple modes of GS action on a Gram-positive bacterial cell. (1) Destabilization/depolarization/osmotic bursting of plasma membrane^51,52,62^; (2) membrane permeabilization for ions and small molecules^39,50,53,57^; (3) inhibition of bacterial respiratory proteins^80^; (4) delocalization and/or clustering of peripheral membrane proteins^57,58^; (5) complexation with nucleic acids^59^ and nucleotides^29,60^; and (6) binding of intracellular signalling molecules^29,61^.

Fortunately, the properties and bactericidal mechanisms of GS have been widely studied in recent decades, and interestingly some multiple targets besides the lipid bilayer have been confirmed (Fig. 8). In particular, these studies revealed that GS is not only able to disrupt lamellar lipid bilayes by forming pores and osmotically bursting the bacterial plasma membrane, but it is also able to directly modulate membrane proteins in various ways^29,39,50–53,57–61^. It was shown, e.g., that GS induces clustering of the membrane proteins MinD and DivIVA, which are involved in cell division. Complete detachment of the phospholipid synthase PlsX, peptidoglycan synthesis enzyme MurG, and cytochrome C (but interestingly not of ATP synthase) were observed in the membranes of *Bacillus subtilis* after GS treatment^57^. Interference with the biosynthesis of phospholipids and peptidoglycan could additionally explain the appearance of the mesosome-like and non-membranous intracellular structures that we observed earlier in the cell interior of GS-treated *S. aureus*^62^.

In addition to this repertoire, we can demonstrate that GS is able to enter the cells of Gram-positive enterococci and staphylococci (SI Fig. S7), acting therefore like a genuine cell-penetrating peptide that enters the cell directly through the plasma membrane (i.e. non-endocytotically)^63^. This intrinsic cell-uptake activity has already been explored in eukaryotic cells to specifically target mitochondria with GS derivatives^64^. In the context of prokaryotic cells, together with the ability to bind certain nucleotides and small phosphorylated molecules^29–33^, GS thus seems to be capable of effectively inhibiting bacterial stress response and biofilm development in a fashion previously attributed to IDR. The periplasmic and intracellular binding of anionic phosphorylated metabolites by GS should deplete their levels, and therefore inhibit metabolic activity and possibly even affect gene regulation, e.g., due to interference with phosphorylation in signal transduction processes. Notably, GS has a very high biostability being cyclic and abundant in non-canonical amino acids. Only one natural non-specific intracellular serine protease, subtilisin (isolated from *B. subtilis*) was shown to be effective in degrading GS^65^. This unique proteolytic stability should effectively prolong any factual activity of GS, e.g. its long-term intracellular binding of nucleotides, and should thus compensate for its slightly inferior binding efficiency compared to the easily degradable IDR or TL, for which the ability to enter bacterial cells is unknown.

Notably, despite long medicinal use, no resistance to GS has ever been documented. We believe that the above-described, multifaceted action of GS strongly impedes the development of acquired resistance. Multiple mechanisms of cell killing by GS can also explain its pronounced synergistic effect in combination with polymyxin B (PMB, which is known to disrupt the outer bacterial membrane by binding to lipopolysacharides) towards biofilms of *P. aeruginosa*^34^. This synergy, observed *in vitro*, suggests the use of GS-PMB combinations to treat infections caused by both *S. aureus* and *P. aeruginosa*^66,67^. As an exemplary case study, we have recently demonstrated the high therapeutic potential of GS and its combination with PMB for the topical treatment of root canal infections^29^.

With its very cheap production costs, gramicidin S may be extremely useful in both households and clinics as an over-the-counter antibacterial and disinfection agent for the prophylactic treatment of primary surface infections. Clinical approval would be timely for numerous applications, e.g. solutions for ear infections, sprays for applying to large burned areas, and ointments or gels for the local treatment, e.g., of post-operative scars or root canal infections^29^.

## Materials and Methods

### Peptide synthesis and purification

GS was produced by fermentation of *Aneurinibacillus migulanus* (DSM 5759; formerly *Bacillus brevis*) and isolated as previously described^68^. For control experiments and specifically to monitor cell uptake, GS and its photoswitchable analogue GS-sw(FP), were also synthesized with standard *N*-Fmoc protocols using peptide synthesizer (Syro II, MultiSynthech) as were TL and IDR. All peptides were characterized by MALDI mass spectrometry (Bruker Autoflex III). Peptides were purified to >95% employing C_18_ columns (Vydac) and water-acetonitrile gradients on a Jasco HPLC system equipped with an ultraviolet diode array detector.

### ^31^P-NMR spectroscopy

Fresh solutions of peptides and P-metabolites in 10% D_2_O (pH 7.2) were combined (1:1 mol:mol with 0.224 mM concentration of each component) directly in 5 mm NMR tubes. Room-temperature proton-decoupled single-pulse ^31^P-NMR spectra (7 µs, delay 2 s) were acquired on a Bruker AVANCE 400 spectrometer (^31^P frequency = 162 MHz) using a Bruker BB-PABBO probe.

### Electrochemical measurements

The setup for the electrochemical characterization of peptide interactions with DOPC-coated Pt/Hg electrodes was performed as previously detailed^69,70^. Current vs. potential RCV scans were performed at a scan rate of 40 V/s by cycling the potential from −0.4 to −1.2 V with a 5 min sampling time. The capacitance current peak at the most negative potential was used for estimating the limit of detection by extrapolating a linear fit of capacitance current peak height vs. peptide concentration (1, 5 and 10 µM).

### Biomimetic chromatography

HPLC was performed on an Agilent 1100 system with an ultraviolet diode array detector. Lipophilicity was measured using a Phenomenex Gemini NT C_18_ column (50 × 3 mm), a 0.01 M formic acid (pH 2.6) or 50 mM ammonium acetate buffers (pH 7.4 and 10.5) as eluent A, and acetonitrile as eluent B. The retention times were standardized and chromatographic hydrophobicity index values were determined and converted to a 1-octanol/water log D scale as described previously^71^. Binding measurements were performed using Regis Technologies IAM.PC.DD2 (100 × 4.6 mm), HiChrom Chiralpak-HSA (50 × 3 mm) and HiChrom Chiralpak-AGP (50 × 3 mm) columns. As eluent A, 50 mM ammonium acetate (pH 7.4) was used with a 0–90% acetonitrile gradient (IAM), or a 0–35% 2-propanol gradient for (HSA and AGP) was employed. Retention times were converted to log k and % binding, using calibration routines described in^72^.

### Haemolysis assay

Haemolytic activity was determined with a serial 2-fold dilution assay. Citrate phosphate dextrose-stabilized human erythrocyte suspensions were obtained from Karlsruhe Municipal Hospital. The washed 0.25% suspension of erythrocytes were incubated with peptides at 37 °C for 30 min as described earlier^33^. The mixture was pelleted (13 000 rpm, 10 min, 4 °C), and the absorbance of the supernatant was recorded at 540 nm vs. reaction buffer. The extent of haemolysis was related to the action of 0.1% Triton X-100 (100%).

### Acute toxicity in *Danio rerio* embryos

The experiment design complied with European Legislation for the Protection of Animals used for Scientific Purposes (Directive 2010/63/EU), in particular, all experiments were planned to be done strictly on larvae below 120 hours post fertilization (hpf), which does not require ethical commission approval. Experiments were performed in accordance with the relevant OECD testing guidelines^73^ and German animal protection standards. Wild type *D. rerio* embryos were obtained at the European Zebrafish Resource Center (Karlsruhe). Fertilized eggs (6 hpf) were raised in 4 ml of embryonic medium (E3) at 28 °C until 72 hpf (6-well plate, 20 eggs/well). Embryos were transferred into flat-bottom glass vials (E3, 1 ml, 15–20 embryos/vial). Peptides were added as 20 µl aliquots in 10% dimethyl sulfoxide in serial 2-fold dilutions. Toxicity was expressed as the concentration at which 50% of the embryos were visually observed to die (measured at 1 and 3 hours exposure) by identifying the heart beat.

### Bacterial strains

Control strains *S. aureus* DSM 1104 and *E. faecalis* DSM 2570 were purchased from the German Collection of Microorganisms and Cell Cultures. The MRSA strains were obtained from the Centre of Medical Microbiology (Karlsruhe Municipal Hospital). The WW4 and WW6 isolates were a kind gift of William G. Wade (King’s College London). The isolates of *E. faecalis* TRE1, TRE2, TRE4 and of *E. faecium* VRE1, VRE2 were obtained from Dr. Staber & Kollegen (Heilbronn). The resistance of the clinical isolates to conventional antibiotics was determined in the original laboratories using the VITEK 2 System (bioMérieux).

### Congo red staining

Biofilms grown on HAD and on agar plates were used for Congo red staining. The HADs were placed into 24-well microtiter plates and filled with 1 ml of bacterial suspension from an overnight culture (final OD_550_ = 0.2, Todd-Hewitt broth). After incubation (37 °C, 24 hours, no agitation), the discs were reintroduced into the wells and exposed to 1 ml of 0.08% aqueous Congo red (Sigma-Aldrich) solution for 2 hours. The results were documented after aspiration of the staining solutions. Bacterial colonies were grown on brain heart infusion agar supplemented with 0.8 mg/ml Congo red but lacking glucose^74^.

### Determination of the minimum inhibitory and bactericidal concentrations

For the determination of MIC, the standard broth microdilution procedure^45^ was used with a slight modification^29^. Since the peptide stock solutions were prepared in 50% ethanol, the first rows of the 96-well microtiter plates were filled with 50 µl of 2 × concentrated Mueller-Hinton broth to reestablish the normal broth concentration after the addition of 50 µl peptide solutions. The MIC was determined as the lowest concentration that inhibited bacterial growth, judging from bacterial respiration in three parallels for each antibiotic. After incubation of the plates for 22 hours, the redox indicator resazurin (Sigma-Aldrich) was added to the wells (20 µl, 80 µM). The plates were incubated another 2 hours at 37 °C and 5% CO_2_. Respiration was determined as the difference in the absorption at 570 nm to 600 nm, using a microplate reader FLASHScan 550 (Analytik Jena).

MBC was determined directly after the MIC evaluation. The 10 µl samples of all 8 dilution rows in the microtiter plates were spotted on square Mueller-Hinton agar plates and incubated overnight at 37 °C and 5% CO_2_. The MBC was determined as the concentration at which no bacterial colonies were obtained on the two parallel spotted plates.

### Time-dependent killing effect of peptides

Killing activity was monitored during 60 min exposure to peptides at concentrations of 5 × and 10 × MIC. Stationary cultures were diluted in Mueller-Hinton broth to OD_550_ = 0.2. The cells were incubated in 4 ml culture tubes at 37 °C with agitation at 220 rpm. Aliquots (100 µl) were taken at 0, 20, 40 and 60 min and diluted 1:10 in Mueller-Hinton broth. Undiluted and diluted aliquots each were each spotted as ten 10 µl spots on the agar plates according to the drop-plate method^75^. Bacterial growth was evaluated by counting colonies on the two parallel spotted plates.

### Determination of the biofilm-forming capacity

Three nutrient media were used: Todd-Hewitt broth, Mueller-Hinton broth and minimal mineral medium^38^, containing 62 mM potassium phosphate (pH 7.0), 7 mM (NH_4_)_2_SO_4_, 2 mM MgSO_4_, 10 µM FeSO_4_, 0.4% glucose and 0.5% casamino acids. The central 6 × 3 wells separated by empty columns in 96-well microtiter plates were filled with overnight bacterial suspensions of OD_550_ = 0.2, and incubated at 37 °C and 5% CO_2_ without agitation for 24 hours. The adherent biofilms were washed, dried, fixed in MeOH, and dried again. Staining was carried out according to^76^ in 100 µl of aqueous 0.1% Crystal violet (Sigma-Aldrich) for 20 min. Excess dye was removed with water, and the wells were dried. The dye absorbed by biofilms was extracted with absolute ethanol (200 µl /well) and quantified in a microtiter plate reader at 595 nm.

### Determination of the minimum biofilm inhibitory concentration

Minimum biofilm inhibitory concentrations causing a 90% decrease in biofilm growth (MBIC_90_) were determined using the microdilution procedure in the same way as for determining MIC, but the wells were inoculated with bacterial cells from stationary cultures with 5 × 10^7^ CFU/ml. In contrast to the determination of MBIC for IDR in^38^, we used Todd-Hewitt broth as a medium. After co-incubation with peptides at 37 °C and 5% CO_2_ without agitation for 24 hours, the planktonic cells were washed, and Crystal violet staining was applied^76^.

### Biofilms on hydroxyapatite discs: regrowth and scanning electron microscopy

Biofilms were grown on standardized hydroxyapatite discs (3D Biotek). The discs were placed in 24-well microtiter plates and inoculated with a cell suspension from the overnight stationary culture, grown in Todd-Hewitt broth and diluted to OD_550 = _0.2. After 30 hours of growth at 37 °C without agitation, the discs were placed for 18 hours in solutions containing 400 µg/ml of peptides, dissolved in 10% dimethyl sulfoxide and 150 mM sodium phosphate buffer (pH 7.2). For the control experiments and conventional antibiotics, pure sodium phosphate buffer was used. After the treatment, discs were washed with sodium phosphate buffer, placed into 1 ml of Todd-Hewitt broth and incubated at 37 °C with agitation at 200 rpm for 24 hours. After the regrowth experiment, aliquots of the culture were spotted on the agar plates as described above to determine the number of remaining viable cells by the negative regrowth.

The second series of hydroxyapatite discs biofilms after the peptide treatment were washed (1x sodium phosphate buffer), fixed in 2% glutaraldehyde (sodium phosphate buffer, 1 hour), washed again (2x H_2_O) and dried under ambient sterile conditions. The samples were sputtered to obtain a 1 nm Pt layer using the high-vacuum coating system EM MED020 (Leica Microsystems). The images were obtained with a Supra 55 VP field emission scanning electron microscope (Carl Zeiss), with an acceleration voltage of 5 kV, working distances of 3.5–3.8 mm, and using an Inlens detector for clear topographic imaging in high vacuum mode, with the chamber pressures of about 2 × 10^−6^ mbar.

### Fluorescent staining

The intrinsic fluorescence of the photoswitchable analogue GS-sw(FP), described previously^77^, was used to study GS translocation into the cytoplasm. Approximately 1 ml of an *E. faecalis* cell suspension (OD_550_ = 1.0) was co-incubated with 100 µg/ml GS-sw(FP) in order to observe cell uptake. Carboxyfluorescein succinimidyl ester (Sigma-Aldrich), which is unable to cross the plasma membrane, was applied for comparison to stain only the extracellular layers and outer  membrane leaflet. The bacterial suspension for the latter staining was resuspended in fresh 150 mM NaHCO_3_ buffer (pH 8.3). At this increased pH, the amino groups of membrane proteins remain deprotonated and can react with the dye. The dye concentration was 1 µl/ml (stock solution 10 mg/ml in dimethyl sulfoxide). Both staining experiments were carried out at 37 °C for 30 min without agitation. Fluorescence was observed using a Axioskop 40 light microscope (Carl Zeiss) equipped with an “A-Plan” objective (100x/1.25 Ph3), a fluorescence filter (type 09, λ_ex_ = 450–490 nm, λ_em_ = 515 nm), and a digital camera (PowerShot G5, Canon). Due to the relatively low fluorescence intensity of GS-sw(FP), the exposure time was increased fourfold for the GS analogue compared to the control.

## Supplementary information

Supplementary information

## Data Availability

The data sets generated and/or analyzed in this study are available from the corresponding author on reasonable request.
