# Supplementary material for: Supreme activity of gramicidin S against resistant, persistent and biofilm cells of staphylococci and enterococci
Source: Sci Rep. 2019 Nov 29;9:17938. doi: 10.1038/s41598-019-54212-z (PMC6884456; doi:10.1038/s41598-019-54212-z)
Supplement: Supplementary file 1 — Supplementary information [file 41598_2019_54212_MOESM1_ESM.pdf]

Supplementary information

Supreme activity of gramicidin S against resistant, persistent and biofilm cells of staphylococci and enterococci

Marina Berditsch[1], Sergii Afonin[2], Jennifer Reuster[1], Hannah Lux[1], Kristina Schkolin[1], Oleg Babii[2], Dmytro S. Radchenko[3,4], Issah Abdullah[5], Nicola William[6], Volker Middel[7], Uwe Strähle[7], Andrew Nelson[6], Klara Valko[5] and Anne S. Ulrich[1,2,*]

[1]Karlsruhe Institute of Technology (KIT), Institute of Organic Chemistry (IOC), Karlsruhe, 76131, Germany;
[2]KIT, Institute of Biological Interfaces (IBG-2), Karlsruhe, 76021, Germany;
[3]Enamine Ltd., Kyiv, 02094, Ukraine;
[4]Taras Shevchenko National University of Kyiv, Kyiv, 01601, Ukraine;
[5]University College London (UCL), UCL School of Pharmacy, London, WC1N 1AX, United Kingdom;
[6]University of Leeds, School of Chemistry, Leeds, LS9 2JT, United Kingdom;
[7]KIT, Institute of Toxicology and Genetics (ITG), Eggenstein-Leopoldshafen, 76344, Germany;

*Anne.Ulrich@kit.edu

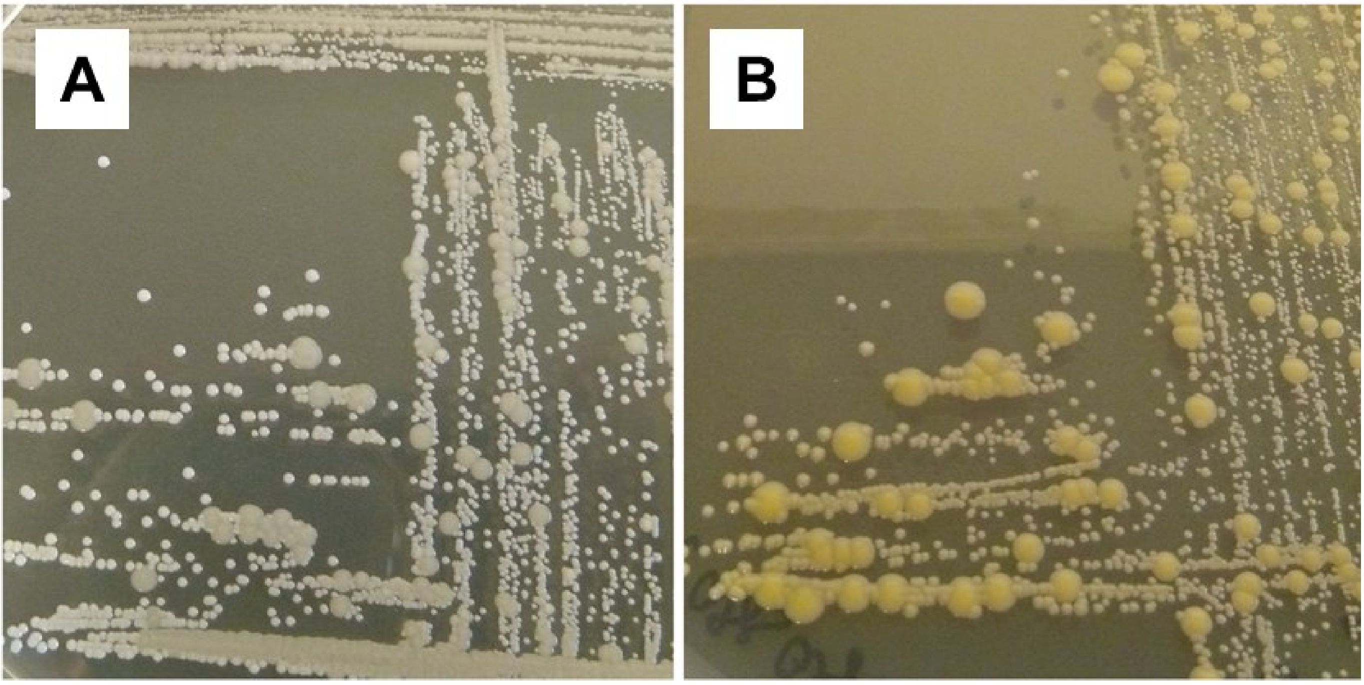

SI Figure S1. Reverse transition of S. aureus SCVs to the classical LCV on TH agar. *(A) –DSM 1104 SCV;* ***(B)*** *– MRSA9 SCV.*

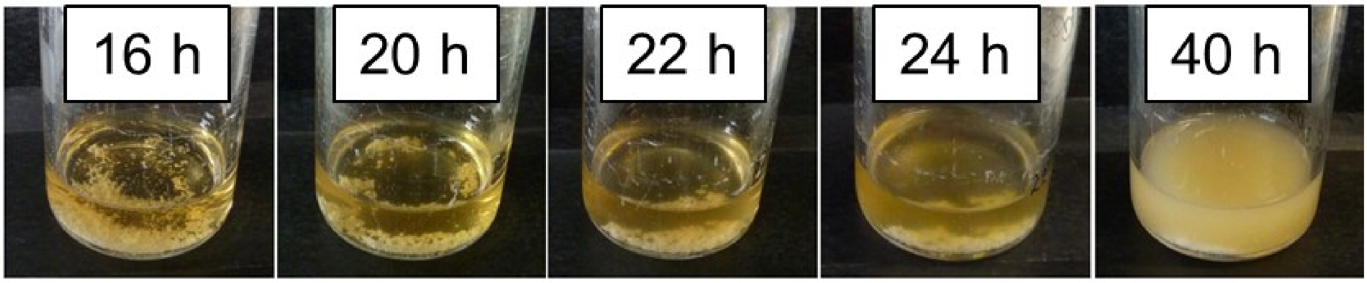

SI Figure S2. Time-dependent reversion from growing as cell agglomerates into common planktonic growth*, observed in the DSM 1104 SCV liquid culture.*

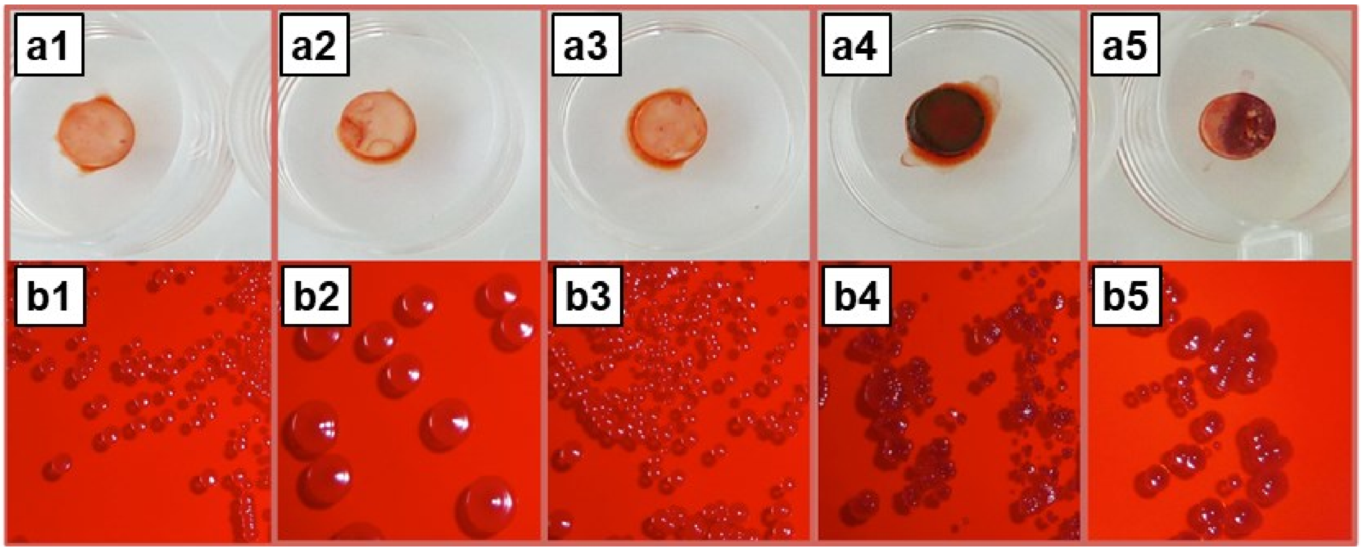

SI Figure S3. Detection of PIA production by Congo red staining. *(top row) Staining of surface-attached biofilms (24 hours growth on HAD in TH broth); (bottom row) Colony staining on brain heart infusion agar. In both cases, aqueous 0.08 % Congo red was used. The strains are designated with the following indeces:* ***a1/b1*** *MRSA8 SCV;* ***a2/b2*** *MRSA9;* ***a3/b3*** *MRSA9 SCV;* ***a4/b4*** *DSM 1104 SCV;* ***a5/b5*** *DSM 1104.*

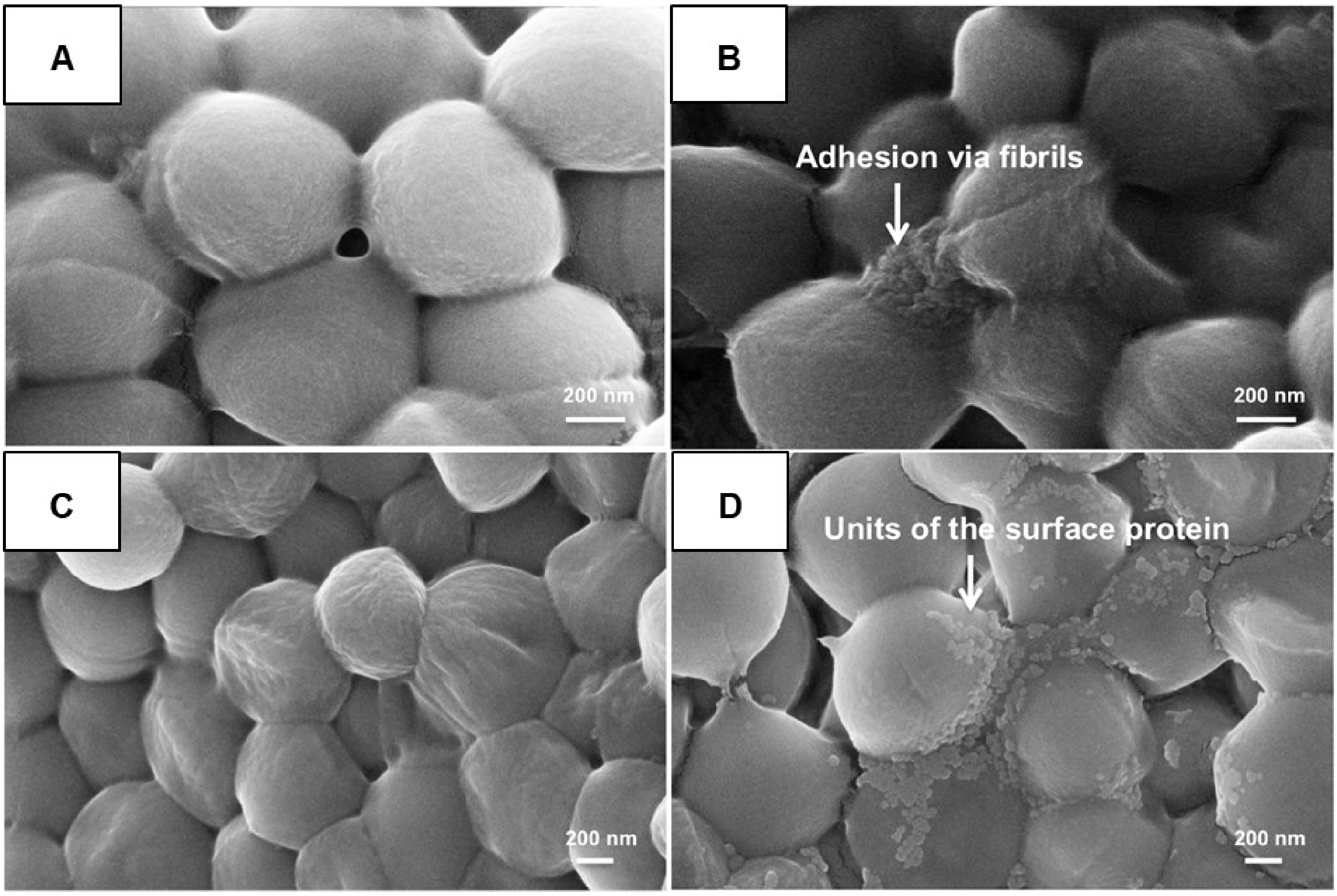

SI Figure S4. SEM images of DSM 1104 SCV biofilm. *(****A*** *and* ***B****) Intact biofilm showing cells being covered by a glazed PIA layer, which attaches them together; (****C****) biofilm after a single re-suspending procedure - the outer PIA layer is lost exposing a deeper proteinous layer; (****D****) the second mechanical wash causes a further loss of surface protein - only several protein units remain visible on the cell surface. Magnification in (****A****) and (****B****) is 50000×, in (****C****) and (****D****) it is 30000×.*

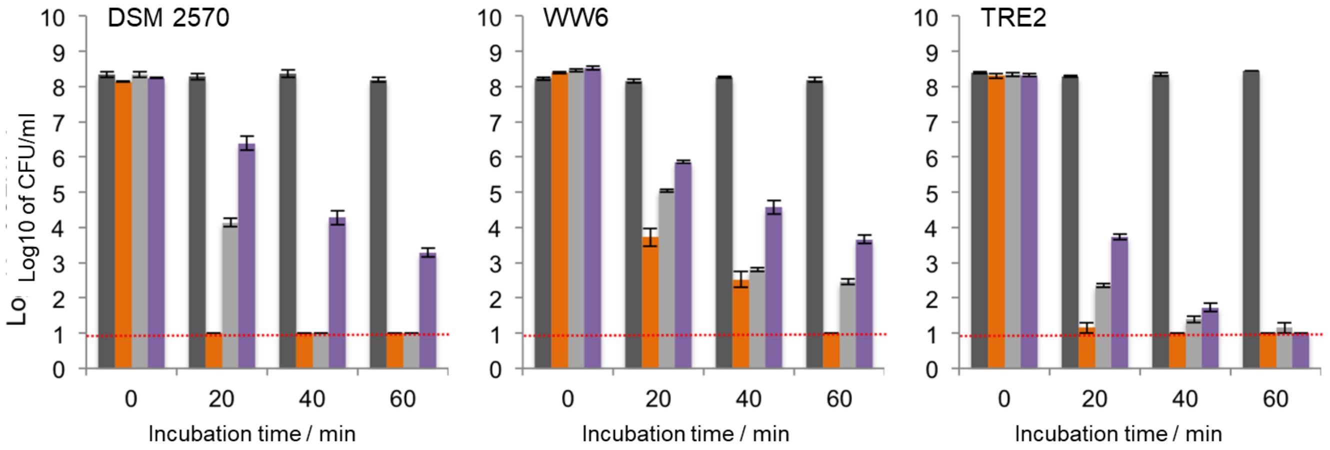

SI Figure S5. Reduction of the cell number under peptide treatment of E. faecalis at 5×MIC: *(A) DSM 2570; (**B**) WW6; (**C**) TRE2. Dark grey bars represent the control (no peptide); cell counts after incubation with GS (orange), TL (light grey) and IDR (purple). The limit of detection (red line) was* 10^1 *CFU/ml.*

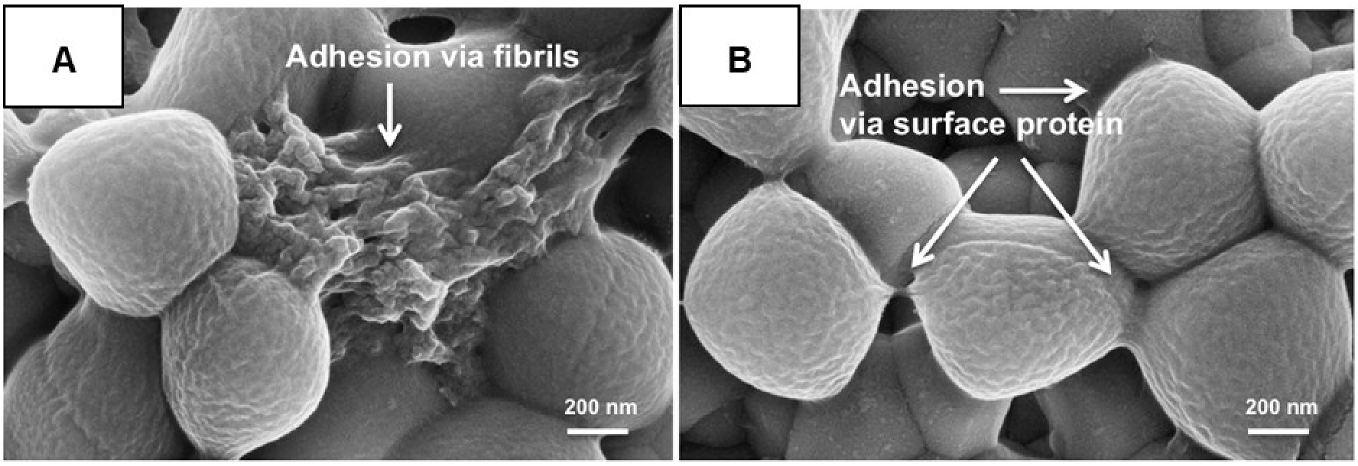

SI Figure S6. Adhesion of MRSA8 SCV clinical strain, isolated from a chronic wound. *(A) Adhesion via fibrils, presumably composed of eDNA; (**B**) adhesion to hydroxyapatite particles and other cells by means of surface protein. SEM, magnification 50000×.*

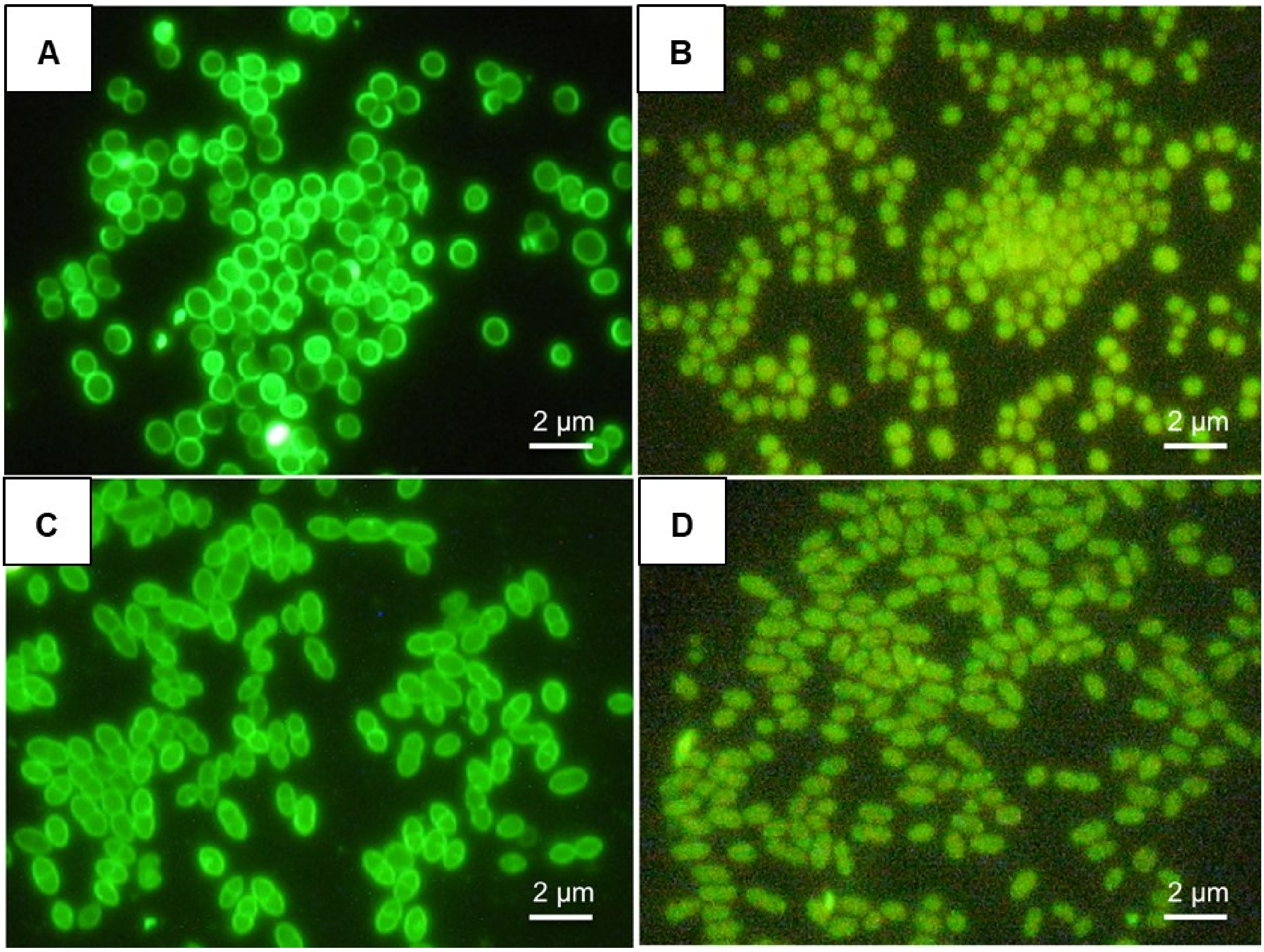

SI Figure S7. Comparative fluorescence microscopy. *Fluorescein is not able to transverse the plasma membrane of (**A**) DSM 1104 SCV and (**C**) E. faecalis TRE2, therefore only surface proteins are labelled. In contrast, the fluorescent GS analogue GS-sw(FP) penetrates the cells of (**B**) DSM 1104 SCV and (**D**) E. faecalis TRE2, as revealed by homogenous fluorescence of the cytoplasm.*

SI Table S1. Studied Strains and their Resistance to Conventional Antibiotics

Strain	Resistance pattern[1]	MDR[2]	Comment
	S. aureus		
DSM 1104	**Sensitive**	**N**	*Control strain*
DSM 1104 SCV	**Sensitive**	**N**	*Laboratory isolate*
MRSA3	**MET, AMP, OXA, TET**	**N**	*Nasal cavity isolate*
MRSA4	**MET, AMP, OXA, GEN**	**N**	*Nasal cavity isolate*
MRSA8 SCV	**MET, AMP, OXA**	**N**	*Wound isolate*
MRSA9	**MET, AMP, OXA, CIP, ERY, CLI, fusidic acid**	**Y**	*Nasal cavity isolate*
MRSA9 SCV	**MET, AMP, OXA, CIP, ERY, CLI, fusidic acid, mupirocin**	**Y**	*Nasal cavity isolate*
MRSA538 SCV	**OXA, AMP, CXM, TOB, CIP, LVX, MXF, CLR, ERY, CLI**	**Y**	*Wound isolate*
	E. faecalis		
DSM 2570	**TET, DMC, GEN**	**N**	*Control strain*
WW4	**TET, DMC, GEN**	**N**	*Cheese isolate*
WW6	**GEN**	**N**	*Root canal isolate*
TRE1	**TET, DMC, CXM, CLI, TMP**	**Y**	*Clinical isolate*
TRE2	**TET, DMC, CXM, GEN, CIP, LVX, MXF, CLR, ERY, CLI, TMP**	**Y**	*Clinical isolate*
TRE4	**TET, DMC, CXM, CLR, ERY, CLI, TMP**	**Y**	*Clinical isolate*
	E. faecium		
VRE1	**VAN, AMP, PIP, CXM, IMP, CIP, LVX, MXF, CLR, ERY, CLI, TMP**	**Y**	*Clinical isolate*
VRE2	**VAN, AMP, PIP, CXM, IMP, CIP, LVX, MXF, CLR, ERY, CLI, TMP**	**Y**	*Clinical isolate*

[1]standard abbreviations of antimicrobial agents are used; [2]"N" =no multidrug-resistance, "Y" =multi-drug resistance;
